# MiR-372-3p Functions as a Tumor Suppressor in Colon Cancer by Targeting MAP3K2

**DOI:** 10.3389/fgene.2022.836256

**Published:** 2022-03-30

**Authors:** Yana Li, Fuqiang Li, Chang Feng, Tingting Wu, Yuyang Chen, Junaid Ali Shah, Fei Wang, Yong Cai, Jianfeng Wang, Jingji Jin

**Affiliations:** ^1^ School of Life Sciences, Jilin University, Changchun, China; ^2^ Department of Ophthalmology and Otorhinolaryngology, Changchun Children’s Hospital, Changchun, China; ^3^ School of Pharmacy, Changchun University of Chinese Medicine, Changchun, China; ^4^ Department of Radiotherapy, China-Japan Union Hospital, Jilin University, Changchun, China

**Keywords:** colon cancer, microRNA, MiR-372-3p, MAP3K2, MAPK signal pathway

## Abstract

MicroRNAs (miRNAs) as small non-coding RNA transcripts bind their complementary sequences in the 3′-untranslated region (3′-UTR) of target messenger RNAs (mRNAs) to regulate their expression. It is known that miR-372 belongs to the miR-371–373 gene cluster and has been found to be abnormally expressed in a variety of cancers, but its precise mechanism in cancer remains to be discovered. In this study, miR-372-3p expression was assessed in 153 frozen tissue samples, including primary diagnosed colon cancer and matched normal and adjacent tissues, using real time quantitative polymerase chain reaction (qPCR). An analysis of qPCR data revealed a significant reduction in miR-372-3p expression (by >2-fold) in colon cancer tissues in 51.5% (34/66) of patients. Consistent with this, mimicking the increased miR-372-3p levels in SW480 colon cancer cells significantly suppressed cell growth and proliferation. Although no direct correlation was found between the low level of miR-372-3p and certain tumor-related factors, such as p53, HRE-2, PMS2, MLH1, MSH2, MSH6, HDAC4, p21, and Wee1, in colon cancer tissues, an inverse relationship between miR-372-3p and Ki67 (a marker of proliferation) or miR-372-3p and MAP3K2(MEKK2), which plays a critical role in the MAPK signaling pathways, was confirmed using tissue samples. The target relationship between miR-372-3p and MAP3K2 was verified using luciferase assays in SW480 colon cancer cells. As expected, miR-372-3p mimics significantly suppressed the luciferase activity of pMIR-luc/MAP3K2 3′-UTR in cells, suggesting that miR-372-3p modulates the expression of MAP3K2 by directly targeting its 3′-UTR. Overall, the results obtained herein suggest that miR-372-3p may function as a tumor-suppressor miRNA in colon cancer by targeting MAP3K2.

## Introduction

MicroRNAs (miRNAs) are a class of evolutionary conserved small non-coding RNAs, which bind to the 3′-untranslated region (3′-UTR) of target messenger RNAs (mRNAs) and play important roles in suppressing protein translation or inducing mRNA degradation as post-transcriptional gene regulators (Bartel et al., 2009). It has been known that miR-372, which belongs to the miR-371–373 gene cluster is located on chromosome 19q13.42. Many oncogenic events related to head and neck squamous cell carcinoma are known to reside in this region (Voorhoeve et al., 2006). The miR-371–373 gene cluster, originally identified as a group of the human embryonic stem cell specific miRNAs, has been found to be involved in the stemness maintenance of embryonic stem cells (Suh et al., 2004). In colorectal cancer cells, miR-372 and miR-373 enhance the stemness by repressing the expression of differentiation genes, such as nuclear factor kappa-light-chain-enhancer of activated B-cells and mitogen-activated protein kinase-like protein/the extracellular signal-regulated kinase (Erk) (Wang et al., 2018), suggesting that this gene cluster mediated signaling pathway is associated with the maintenance of stemness. In addition, numerous research data suggest that the miR-371–373 gene cluster is tightly associated with tumorigenesis and progression in various types of human cancers (Shah et al., 2021).

Consistent with this, aberrant expression of miR-371–373 has been found in several primary diagnosed types of cancers. It is worth noting that the expression levels of miR-371–373 vary according to the type of cancer. For example, miR-372–373 expression is significantly lower in non-small cell lung cancer (Seol et al., 2014), pancreatic cancer (Nakata et al., 2014), prostate cancer (Kong et al., 2016), cervical cancer tissues (Tian et al., 2011), endometrial cancer (Liu et al., 2016), ovarian carcinoma (Guan et al., 2017), osteosarcoma tissues (Xu et al., 2018) and renal cell carcinoma tissues (Huang et al., 2015), compared to in normal tissues. Conversely, in some tumor tissues, miR-372 was more highly expressed than in normal tissues, including in lung squamous cell carcinoma (Wang et al., 2017), colorectal carcinoma tissues (Yu et al., 2016), esophageal squamous cell carcinoma (Ghasemi et al., 2018), oral squamous cell carcinoma (Tu et al., 2015), hepatocellular carcinoma (Gu et al., 2013), and glioma (Li et al., 2013). It is worth mentioning that the expression results of miR-372 in breast cancer tissues reported by two different groups are in opposition: although low expression of miR-372 in 20 primary breast cancer tissues compared to paired normal tissues was documented by Liu et al. (Zhao et al., 2017), higher expression of miR-372 in the same type of cancer tissues was observed by Chen et al. (Cheng et al., 2018), suggesting the complexity of the molecular mechanism of miRNAs in tumorigenesis. In other words, the expression status of miRNAs in different cancer tissues may closely correlate with their interacting target genes.

Accumulating evidence has demonstrated that miR-372 is involved in the regulation of a variety of important biological processes in cells, such as cell proliferation, apoptosis, migration, and invasion, in many types of human cancers (Shah et al., 2021). Theoretically, miRNAs bind their complementary sequences in the 3′-UTR of target genes to degrade targeted mRNA by an RNA-interference mechanism. However, in mammalian cells, there is almost no perfect complementarity between miRNAs and protein-coding genes, so it is difficult to directly pinpoint relevant downstream targets of a miRNA (John et al., 2004; Shah et al., 2021). Therefore, it is not difficult to understand that miR-372 can target multiple genes to regulate various intracellular processes. Depending on the genes being targeted, miR-372 may serve oncogenic or suppressive roles in different cancer cells. Oncogenic roles of miR-372 have been reported by several research groups to date. For example, miR-372 facilitates the proliferation of colorectal cancer (Peng et al., 2019), breast cancer (Cheng et al., 2018), and gastric cancer (Cho et al., 2009) cells through binding to target sites in the 3′-UTR of large tumor suppressor homolog 2. Moreover, miR-372 promotes cell proliferation and invasion in both lung squamous cell carcinoma and head and neck squamous cell carcinoma cells through inhibiting fibroblast growth factor 9 and p62, respectively (Yeh et al., 2015; Wang et al., 2017). In contrast, miR-372 acts as a tumor suppressor, inhibiting growth and metastasis in different cancer cells, including osteosarcoma, renal cell carcinoma, prostate cancer, hepatocellular carcinoma, breast cancer, and cervical cancer cells, by targeting and binding to the complementary sequences in the 3′-UTRs of FXYD domain containing ion transport regulator 6 (Xu et al., 2018), insulin-like growth factor 2 mRNA-binding protein 1 (Huang et al., 2015), p65 (Kong et al., 2016), ATPase family AAA domain-containing protein 2 (Wu et al., 2014), E2F transcription factor 1 (hou et al., 2017), and cyclin-dependent Kinase 2/cyclinA1 (Tian et al., 2011), respectively. In endometrial cancer cells, miR-372 inhibits cancer development by targeting the expression of the Ras homolog gene family member C (Liu et al., 2016).

Colorectal cancer is one of the most common malignant tumors in the world. In 2020, approximately 147,950 individuals were diagnosed with colon cancer, and about 36% of patients died from the disease, in the United States (Siegel et al., 2020). According to the published literature, miR-372/373 in colorectal cancer can be trans-activated by wingless (Wnt)/β-catenin signaling, which further upregulates miR-372/373 to contribute to cancer stem cell like properties (Zhou et al., 2012; Wang et al., 2018). However, it is unclear whether miR-372 is involved in regulating signaling pathways other than the Wnt-signal pathway. In addition, although there have been some reports about the expression of miR-372 in various tumor tissues, none exist regarding its expression in colon cancer only tissues. In this study, we collected and detected the expression level of miR-372-3p in 153 frozen tissue samples (including 66 colon cancer tissues, matched with 66 normal and 21 adjacent tissues), and then analyzed the correlation between miR-372-3p expression levels with pathological immunohistochemical (IHC) staining parameters, including tumor-suppressor gene p53, mutL homolog 1 (MLH1), mutS homolog 2 (MSH2), mutS homolog 6 (MSH6), mismatch repair system component 2 (PMS2), marker of proliferation Ki-67 (Ki67) and hypoxia response element-2 (HRE-2) and several potential miR-372-3p target genes. We found that miR-372-3p mimics inhibit the cell viability and clone-formation ability in SW480 colon cancer cells, and this effect may result from the targeted inhibition of mitogen-activated protein kinase kinase kinase 2 (MAP3K2, MEKK2) expression.

## Materials and Methods

### Tissue Collection

All specimens were handled and made anonymous according to ethical and legal standards. Sixty-six patients with pathologically diagnosed colon cancer were enrolled in this study, and all included patients underwent radical surgery between November 2017 and August 2018 at the China–Japan Union Hospital of Jilin University without receiving any neoadjuvant radiotherapy and chemotherapy before their surgical operation. However, patients who had previously suffered from other malignancies or with diseases of the immune and endocrine system were excluded. The number of registration of Committees approvals for tissue collection is 201707018. The tumor, adjacent (<2 cm away from the tumor area) and normal (>5 cm away from the tumor area) tissues excised during surgery were prepared into tissue blocks. All samples were partially frozen for quantitative polymerase chain reaction (qPCR) detection, and left samples were immediately fixed in 4% formalin for hematoxylin and eosin (H&E) staining and IHC analysis. IHC information included p53, Ki67, HRE-2, MLH1, MSH2, MSH6, and PSM2 as provided by the China–Japan Union Hospital of Jilin University. The median age of the patients was 61 years (range, 30–85 years). Written informed consent was obtained from all participants, and the study was approved by the institutional ethics board of the Jilin University. Details of patient medical records, including patient age and gender, tumor staging, pathological diagnosis, IHC images of tumor-associated proteins, and surgical records were reviewed. Tumors were staged according to the 2010 TNM classification system of the American Joint Committee on Cancer (Edge et al., 2010).

### Antibodies and Reagents

Anti-MAP3K2 (BA3634-2) polyclonal antibody was purchased from BOSTER Biological Technology Co. Ltd. (Wuhan, China). Anti-glyceraldehyde-3-phosphate dehydrogenase (GAPDH) was raised against bacterially expressed proteins (Jilin University, Changchun, China).

### Cell Culture

Human SW480 colon cancer cells were obtained from the Type Culture Collection of the Chinese Academy of Sciences (Shanghai, China). Cells were cultured in Dulbecco’s Modified Eagle’s Medium (DMEM, Sigma-Aldrich, St. Louis, MO) with 5% glucose and 10% fetal bovine serum (KY-01003, Kang Yuan Biology, Tianjin, China), 100 U/mL penicillin, and 100 mg/ml streptomycin (Thermo Fisher Scientific, Waltham, MA, United States) in 10 cm dishes at 37°C in a humidified atmosphere of 5% CO_2_.

### Reverse Transcription PCR

Total RNA was extracted from 1 × 10^7^ cells or tissues using TRIzol® LS reagent (Invitrogen, Carlsbad, CA, United States), and 1 μg of RNA from each sample was used as a template to produce cDNA with the PrimeScript first Strand cDNA synthesis Kit (Takara Bio, Shiga, Japan). miR-372-3p, WEE1 G2 checkpoint kinase (Wee1), histone deacetylase 4 (HDAC4), cyclin dependent kinase inhibitor 1A (p21), MAP3K2, and GAPDH mRNA levels were analyzed by real-time qPCR with the SYBR®Premix EX Taq kit (Takara Bio, Shiga, Japan). The PCR reactions were finished under the following program: initial denaturation at 95°C for 3 min, followed by 35 cycles of denaturation at 95°C for 30 s, annealing at 60°C for 30 s, and extension at 72°C for 30 s. The primer sets used for PCR are listed in Table 1. The expression of miR-372-3p was normalized to U6, and those of other mRNAs were normalized to GAPDH. Three independent experiments with three replicates per group were conducted. The relative expression levels of miR-372-3p, Wee1, HDAC4, p21 and MAP3K2 were calculated using the 2^−∆∆CT^ method.

**TABLE 1:** Sequence primers designed for real-time qPCR.

Genes	Forward	Reverse
miR-372-3p	TAG​CAG​GAT​GGC​CCT​AGA​CC	TCC​GTT​GAT​ATG​GGC​GTC​AC
Wee1	GCT​TGC​CCT​CAC​AGT​GGT​ATG	CCG​AGG​TAA​TCT​ACC​CTG​TCT​GA
HDAC4	GGC​CCA​CCG​GAA​TCT​GAA​C	GAA​CTC​TGG​TCA​AGG​GAA​CTG
p21	ATG​TGG​ACC​TGT​CAC​TGT​CTT​G	CGT​TTG​GAG​TGG​TAG​AAA​TCT​G
MAP3K2	TCT​GTT​TTA​TCT​TCT​CAG​GCC​A	TGC​AAG​GAT​AAT​GCT​GGT​CG
GAPDH	ATC​ACT​GCC​ACC​CAG​AAG​AC	ATG​AGG​TCC​ACC​ACC​CTG​TT
MAP3K2-3′-UTR (21-472)	CGC​TAG​CAC​GCG​TCC​TCT​ACC​TAG	CAG​TAC​CGG​ATT​GCC​AAG​CTT​ACT
MAP3K2-3′-UTR (6394-66)	CTC​GCT​AGC​ACG​CGT​TGA​TGT	AAC​AGT​ACC​GGA​TTG​CCA​AGC​TT
miR-372-3p RT	ACA​CTC​CAG​CTG​GGA​AAG​TGC​TGC​GAC​ATT​T	GTGCAGGGTCCGAGGT
U6 RT	CTC​GCT​TCG​GCA​GCA​CAT​ATA​CT	ACG​CTT​CAC​GAA​TTT​GCG​TGT​C

### Construction of Plasmids

A 341-bp DNA fragment carrying pri-miR-372-3p was inserted between the XhoI and BamHI sites in the pmR-mCherry vector. The 3′-UTR fragments of human MAP3K2 (21 to +472 bp, +6394 to +6656 bp) were amplified by real-time PCR and cloned downstream of luciferase between the MulI and HindIII sites. Similarly, 3′-UTR mutants, which contained mutated miR-372-3p binding sites (AGCACTTT), were cloned to the pMIR-Report-luc between the same sites. The primer sequences used for RT-PCR amplification are shown in Table 1.

### Cell Transient Transfection

Cells cultured in 24-well plates were transfected with pmCherry-miR-372-3p plasmids, miR-negative controls (NCs), or pMIR-Report-Luc/MAP3K2-3′-UTR wild type (WT) or mutant (MT) plasmids using polyethylenimine (23966) (Polysciences, Shenzhen, China). Synthetic miR-372-3p inhibitors (AGA​AUA​GUG​CUC​CAC​AUU​UGA​GG) were transfected with Lipofectamine™ 2000 (Invitrogen, Carlsbad, CA, United States) following the manufacturer’s instructions. At 48 or 72 h after transfection, cells were harvested and lysed in radioimmunoprecipitation assay lysate buffer (1% NP-40, 150 mM of NaCl, 50 mM of Tris-HCl, 10% glycerol, 1 mM of dithiothreitol, and complete protease inhibitor cocktails). Proteins in whole-cell lysates were analyzed by western bloting using specific antibodies.

### Luciferase Reporter Assay

SW480 cells were co-transfected with 0.4 μg of pmCherry–miR-372-3p, or the reporter plasmids described above, which encode firefly luciferase, and the control plasmid Renilla luciferase vector (0.12 ng), which encodes Renilla luciferase. At 24 h after the transfection, the cells were harvested, and the luciferase activity of pMIR-Report-Luc/MAP3K2-3′-UTR was determined by measuring firefly and Renilla luciferase activities using the Dual-luciferase Reporter assay kit (Promega, Madison, WI, United States) and by normalizing to Renilla luciferase according to the manufacturer’s instructions. Three biological replicates were conducted.

### Cell Viability and Growth Assay

Cells were cultured at a density of 3 × 10^3^/well in 96-well plates and treated with miR-372-3p and/or inhibitors following the experimental design. At given time points after transfection (24 and 48 h), cells were incubated with 10 μL of cell counting kit-8 (CCK-8) reagent (017319, Promega Corporation, Madison, WI, United States) for 1 h at 37°C. The absorbance at a wavelength of 450 nm was measured using a microplate reader (Infinite F200 Pro, TECAN, Shanghai, China).

### Colony Formation Assay

SW480 cells grown to ∼30% confluence in 6-well plates were treated with the control vector, pmCherry–miR-372-3p, with or without miR-372 inhibitors Forty-eight hours later, cells were digested with trypsin, re-suspended in Dulbecco’s modified eagle medium, and split into a new 12-well plate with 2 × 10^3^ cells/well. After 7 days of culture, formed colonies were stained with 0.1% crystal violet. Colonies containing >20 cells were scored as positive. Colonies were photographed using the Gel Imaging System (Liuyi Instrument Plant, Beijing, China).

### Statistical Analysis

The data are reported as mean ± standard deviation values. Kolmogorov-Smirnov test were performed to verify the distribution of data. For the comparison of two study groups, statistical analysis was performed using Mann Whitney t-test. Differences among the three groups were analyzed by one-way analysis of variance (ANOVA) using the SPSS version 19.0 software (IBM Corporation, Armonk, NY, United States) or with the GraphPad Prism software (GraphPad Software, San Diego, CA, United States). *p* < 0.05 was regarded as statistically significant.

## Results

### Frequent Downregulation of miR-372-3p Was Observed in Primary Diagnosed Colon Cancer Tissues

**FIGURE 1:**
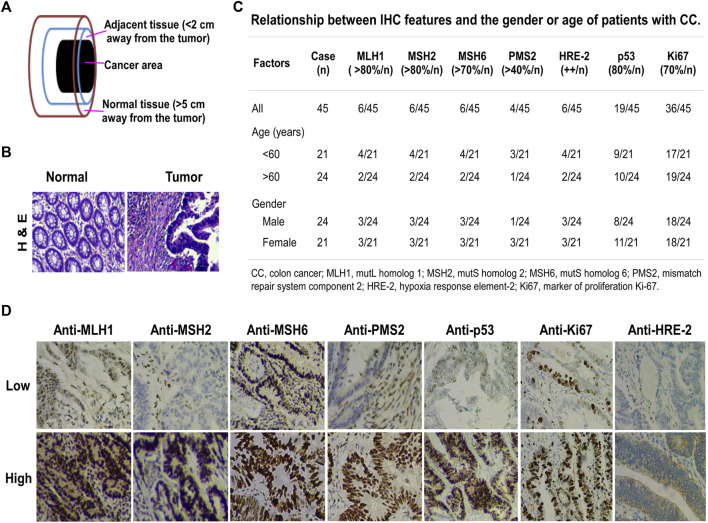
Clinicopathological characteristics of primary diagnosed colon cancer. **(A)** Tissue samples. Cancer area, the site of pathologically diagnosed colon cancer; adjacent tissue, <2 cm away from the tumor in which the cells were pathologically normal; normal tissue, >5 cm away from the cancer. **(B)** H&E staining, magnification ×200. Left, normal tissue; right, colon cancer tissue. **(C)** Relationship between IHC features and the gender or age of colon cancer patients. The percentage indicates the amount of positive IHC staining. The number before the slash in the table represents the number of cases in line with the corresponding positive percentage. **(D)** IHC staining for MLH1, MSH2, MSH6, PMS2, p53, Ki67, and HRE-2 in colon cancer tissues. Upper, IHC low expression; lower, IHC high expression.

To investigate the involvement of miR-372-3p expression in the pathogenesis of primary colon cancer, the tumor, corresponding adjacent, and normal tissues were collected from 66 patients, including 45 with paired primary diagnosed colon cancer and matched normal tissues, and 21 with primary diagnosed colon cancer, matched adjacent and normal tissues (Figure 1A). H&E staining images of tumor and normal tissues taken under a microscope are shown in Figure 1B. In addition, the IHC information of pathological tissue sections of 45 patients with colon cancer was collected, including p53, Ki67, HRE-2, PMS2, MLH1, MSH2, and MSH6; these proteins are used as reference indicators for tumor diagnosis in routine clinical testing (Figure 1C). The numbers in the Figure 1C represent the number of cases of the above IHC-stained proteins in different age and gender groups, and the high or low expression levels of different proteins are detailed in Figure 1D.

**FIGURE 2:**
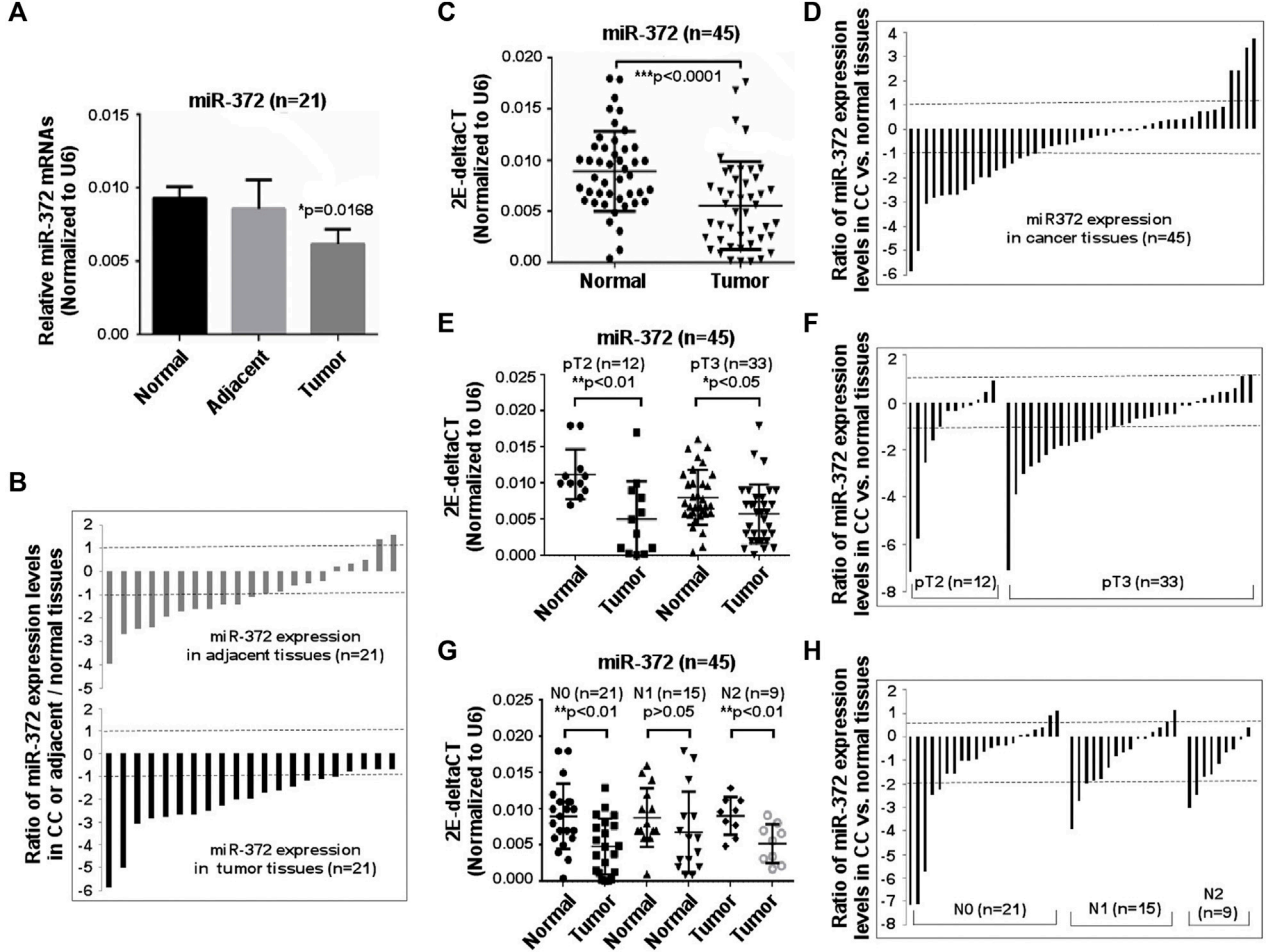
Downregulation of miR-372-3p was observed in colon cancer tissues. **(A)** miR-372-3p expression levels in colon cancer. Tissue samples from 21 colon cancer patients and their matched adjacent and normal tissues were used to assess the miR-372-3p expression (normalized to U6). Data are presented using mean ±standard deviation values. *p < 0.05 vs. normal group. **(B)** Expression patterns of miR-372-3p in colon cancer. Each bar presents the log2 value of the ratio of miR-372-3p expression levels between colon cancer and matched normal tissues or adjacent and normal tissues from the same patients (*n* = 21). A bar value >1 represents a >2-fold increase, whereas a bar value <1, represents a >2-fold decrease. Each bar represents the means of three independent replications. **(C)** 2E-deltaCT (normalized to U6) values of miR-372-3p in colon cancer and normal tissues. ***p < 0.001 vs. normal tissues. **(D)** Ratios of miR-372-3p expression levels between colon cancer and matched normal tissues from the same patients (n = 45). **(E)** 2E-deltaCT analysis of pathologic stage T2 and T3 tissue samples. Values are displayed per 2E-deltaCT (normalized to U6) in colon cancer or matched normal tissues. *p < 0.05, **p < 0.001 vs. normal tissues. **(F)** Patterns of miR-372-3p mRNA expression at pathological stage T2 and T3. **(G)** Results of 2E-deltaCT analysis performed according to the lymph node status. *p < 0.05, **p < 0.001 vs. the normal group. **(H)** Patterns of miR-372-3p mRNA expression in different lymph node status. N0, no nearby lymph node metastasis; N1, one to two nearby lymph node metastasis; N2, three to six nearby lymph node metastasis. All qPCRs were performed in three independent experiments with three replicates per group. Statistical differences between two groups were analyzed using the Mann Whitney test. CC, colon cancer.

To determine whether miR-372-3p is involved in the pathogenesis of primary colon cancer or not, 21 tumor, matched adjacent and normal tissues were first used. As shown in Figure 2A, a statistically significant difference appeared between tumor and matched normal tissues (*p < 0.05). Further analysis of the expression of 21 samples showed significant (>2-fold decreased) downregulation of miR-372-3p in 80.9% (17/21) of patients. Interestingly, miR-372-3p expression in adjacent tissues also exhibited a reduction (>2-fold decrease) in 52.4% (11/21) of samples (Figure 2B). To expand upon the observations given above and to determine the relationship between miR-372-3p expression and clinicopathological parameters, qPCR results were analyzed according to the clinical characteristics of colon cancer. Thus, an additional 45 paired clinical colon cancer and matched normal tissues were used to further validate the frequent downregulation of miR-372-3p expression in primary colon cancer tissues. There was a significant difference in overall miR-372-3p expression between tumor and matched normal tissues (***p < 0.001) (Figure 2C). An analysis of mRNA expression levels of the 45 samples revealed significant (>2-fold decreased) downregulation of miR-372-3p mRNA in 37.8% (17/45) of patients, whereas 8.9% (4/45) of patients showed a significant (>2-fold increased) upregulation of miR-372-3p (Figure 2D). Hereafter, detailed statistical analyses were completed to further explore the correlation between miR-372-3p expression and clinical features. The relationship between miR-372-3p expression and pathological stage revealed significantly low levels of miR-372-3p expression in pT2- (**p < 0.01) and pT3-stage (*p < 0.05) colon cancer compared to normal tissues (Figure 2E). There was significantly less (>2-fold) miR-372-3p in 41.7% (5/12) of pT2-stage and 48.5% (16/33) of pT3-stage tissue samples compared to normal tissues (Figure 2F). Moreover, a significant downregulation of miR-372-3p level in the N0 (**p < 0.01) and N2 (**p < 0.01) of lymph node metastasis groups was observed (Figure 2G). A >2-fold reduction of miR-372-3p was found in 42.9% (9/21) of the N0 group and 55.6% (5/9) of the N2 group (Figure 2H). More detailed relationship between miR-372 expression (qPCR) and clinicopathological characteristics of colon cancer is shown in Supplementary Table S1.

**FIGURE 3:**
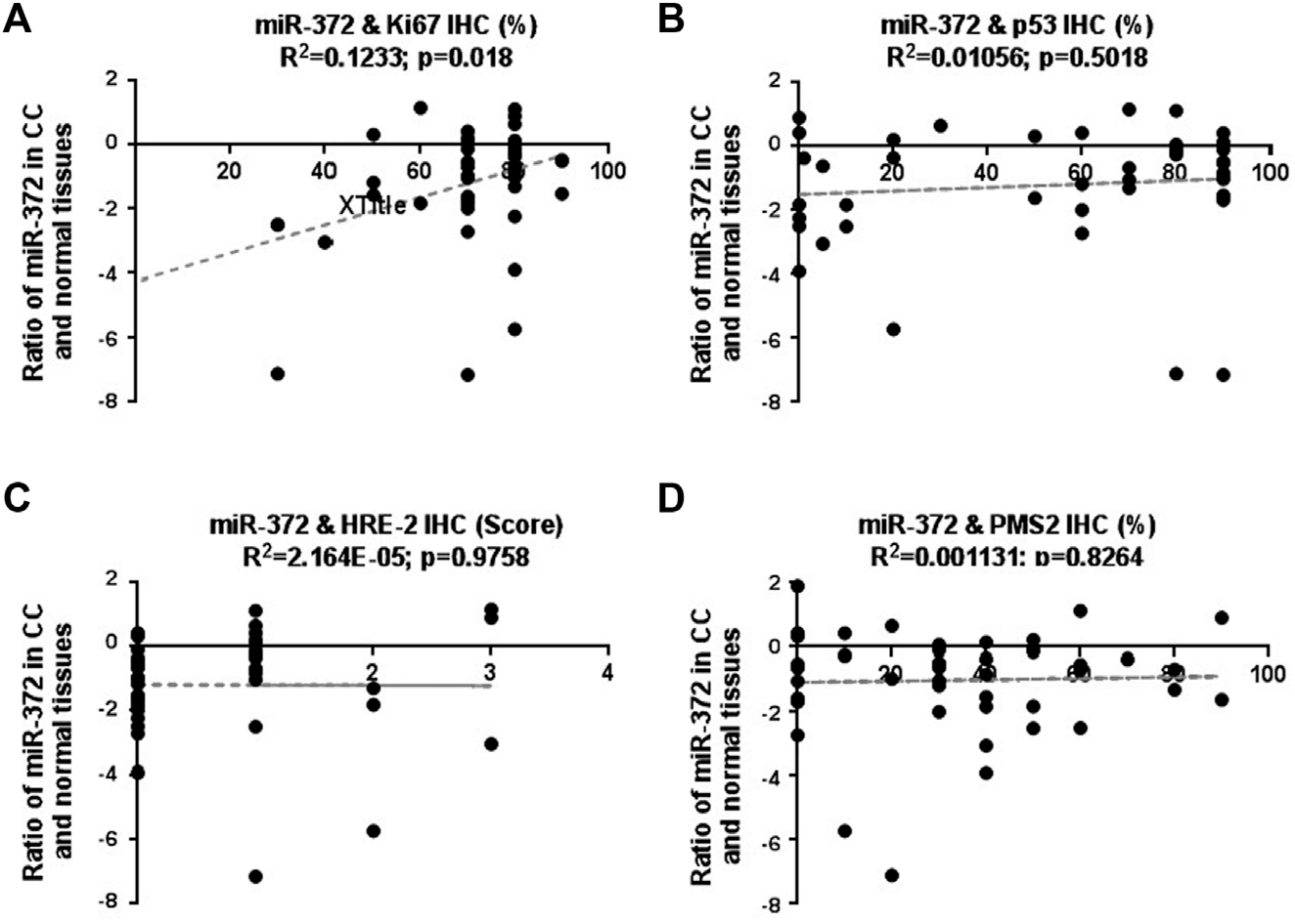
Correlation between miR-372-3p expression and clinicopathological parameters, including Ki67, p53, HRE-2, MSHs, and PMS2, in colon cancer tissues. The Y-axis exhibits the log2 values of the ratio of miR-372-3p expression levels between colon cancer and matched normal tissues, and the X-axis exhibits the positive percentages of IHC staining of Ki67 **(A)**, p53 **(B)**, HRE-2 (scores) **(C)**, MLHs/PMS2 **(D)**. The correlation between miR-372-3p expression and above parameters was statistically analyzed using the GraphPad Prism software.

To explore the correlation between low expression of miR-372-3p and the aforementioned parameters, such as p53, Ki67, HRE-2, MSHs/PMS2, the miR-372-3p expression levels of 45 samples were analyzed according to the different IHC-positive percentages of the above parameters. As shown in Figure 3A, there was a correlation between low expression of miR-372 and highly expressed Ki67 (R^2^ = 0.1233; *p* = 0.018), in other words, low expression of miR-372 was more evident in tissues with high Ki67 expression, suggesting that miR-372 may be involved in cell proliferation. However, no correlation was found between low expression of miR-372 and expression of p53 (R^2^ = 0.01056; *p* = 0.5018), HRE-2 (R^2^ = 2.164E-05; *p* = 0.9758), or MSHs (R^2^ = 0.001131; *p* = 0.8264) (Figures 3B–D). More detailed relationship between miR-372 expression (qPCR) and IHC parameters of colon cancer is shown in Supplementary Table S2.

### Expression of miR-372-3p and the MAP3K2 Gene in Colon Cancer Tissues Was Negatively Correlated

**FIGURE 4:**
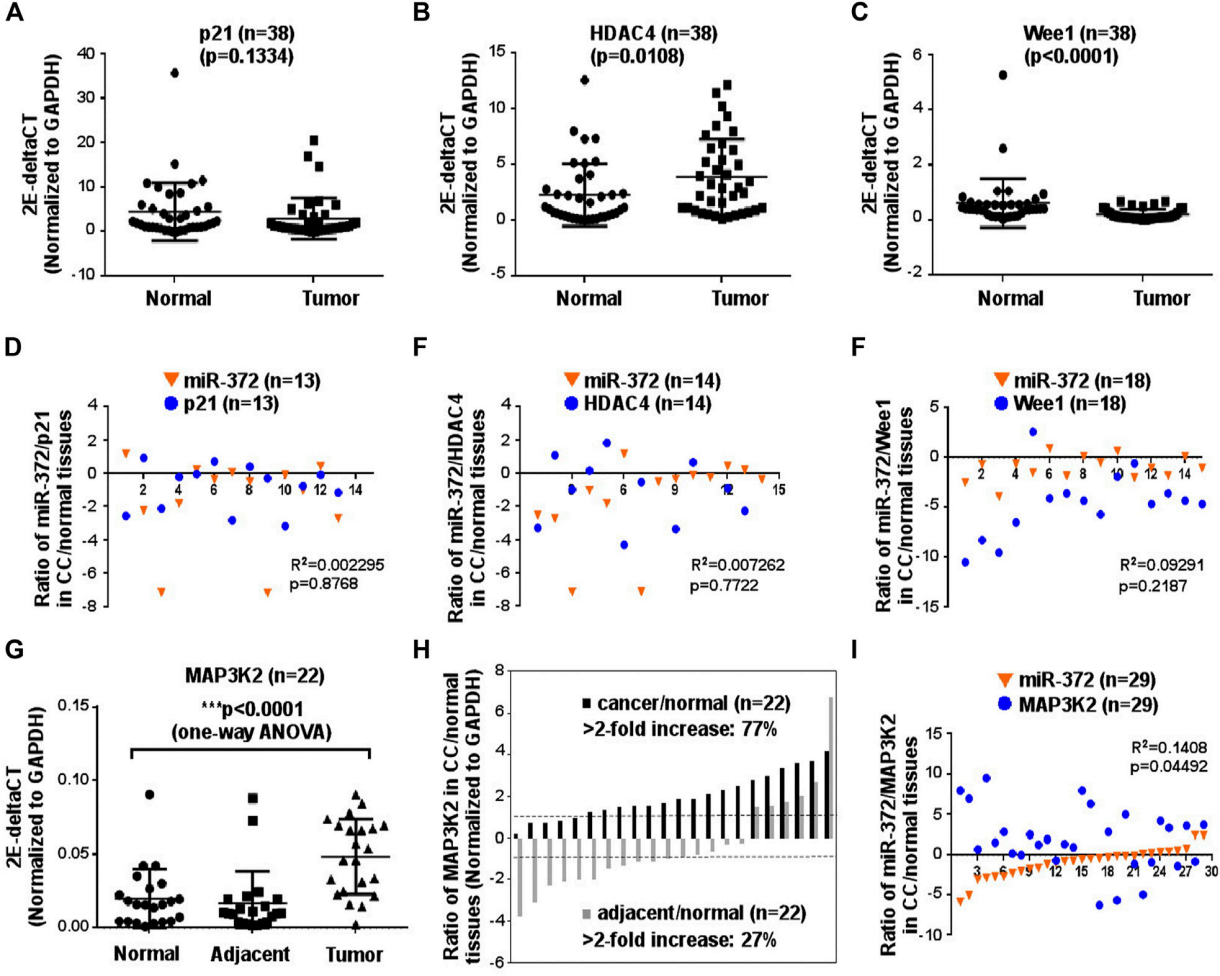
MAP3K2 may be the potential target of miR-372-3p in colon cancer tissues. **(A–C)** mRNA expression levels of the potential targets of miR-372-3p including p21, HDAC4, and Wee1, are displayed as 2E-deltaCT (normalized to GAPDH) in colon cancer or matched normal tissues. **p < 0.01 vs. normal tissues (Mann Whitney test). **(D–F)** Correlation between miR-372-3p expression and its potential targets p21, HDAC4, and Wee1 in colon cancer. The Y-axis exhibits the log2 value of the ratio of miR-372-3p (orange triangle) and p21, HDAC4, or Wee1 (blue circle) expression levels between colon cancer and matched normal tissues, and the X-axis exhibits the number of samples. The correlation between miR-372-3p and p21, HDAC4, or Wee1 was statistically analyzed using the GraphPad Prism software. **(G)** MAP3K2 mRNA expression (normalized to GAPDH) in colon cancer, matched adjacent and normal tissues. ***p < 0.0001 vs. normal tissues (one-way analysis of variance). **(H)** Expression patterns of MAP3K2 in colon cancer and matched adjacent tissues. Each bar presents the log2 value of the ratio of MAP3K2 expression levels between colon cancer and matched normal tissues or adjacent and normal tissues from the same patients. **(I)** Correlation between miR-372-3p and MAP3K2 expression levels in colon cancer tissues. The Y-axis exhibits the log2 value of the ratio of miR-372-3p (orange triangle) and MAP3K2 (blue circle) expression levels, and the X-axis exhibits the number of samples. All qPCRs were performed in three independent experiments with three replicates per group.

To elucidate the potential mechanism of miR-372-3p mediated tumorigenesis in colon cancer, HDAC4, Wee1, p21, and MAP3K2 were selected as the potential targets of miR-372-3p since complementary sequences were found in their 3′-UTR regions. The relative expression levels of HDAC4, p21, and Wee1 were measured using the qPCR approach (Figures 4A–C). Compared to matched normal tissues, no statistically significant difference was observed in the gene expression of p21 in colon cancer tissues (*p* > 0.05). However, higher expressed HDAC4 (*p < 0.05) and lower expressed Wee 1 (***p < 0.0001) mRNA were observed in tumor tissues than that of the matched normal tissues, respectively (Figures 4B,C). To facilitate the observation of the correlation between miR-372-3p and potential genes, the log2 value of the ratio between tumor and matched normal tissues from the same patient was placed on the same chart. Obviously, there was an irregular correlation between the expressions of miR-372-3p and HDAC4 (R^2^ = 0.007252; *p* = 0.7722) or miR-372-3p and p21 (R^2^ = 0.002285; *p* = 0.8768) or miR-372-3p and Wee1 (R^2^ = 0.009291; *p* = 0.2187) (Figures 4D,E). MAP3K2, a member of the serine/threonine protein kinase family, plays a critical role in the MAP kinase signaling pathway by activating other kinases. In our experiments, a significant increase in MAP3K2 expression was observed in colon cancer tissues compared to matched normal tissues (***p < 0.0001) (Figure 4G). Further analysis of the expression of 22 colon cancer samples showed significant (>2-fold increased) up-regulation of MAP3K2 in 77.3% (17/22) of patients, while there was an increase (>2-fold decrease) in 27.3% (6/22) of adjacent tissue samples (Figure 4H). In the scatterplot, the log2 value of the ratio between tumor and matched normal tissues from the same patient seemed to show a negative correlation (R^2^ = 0.1408; *p* = 0.04492), suggesting a potential relationship between MAP3K2 and miR-372-3p (Figure 4I).

### miR-372-3p Mimics Suppressed Cell Proliferation and Negatively Regulated MAP3K2 Expression by Targeting its 3′-UTR Region in SW480 Colon Cancer Cells

**FIGURE 5:**
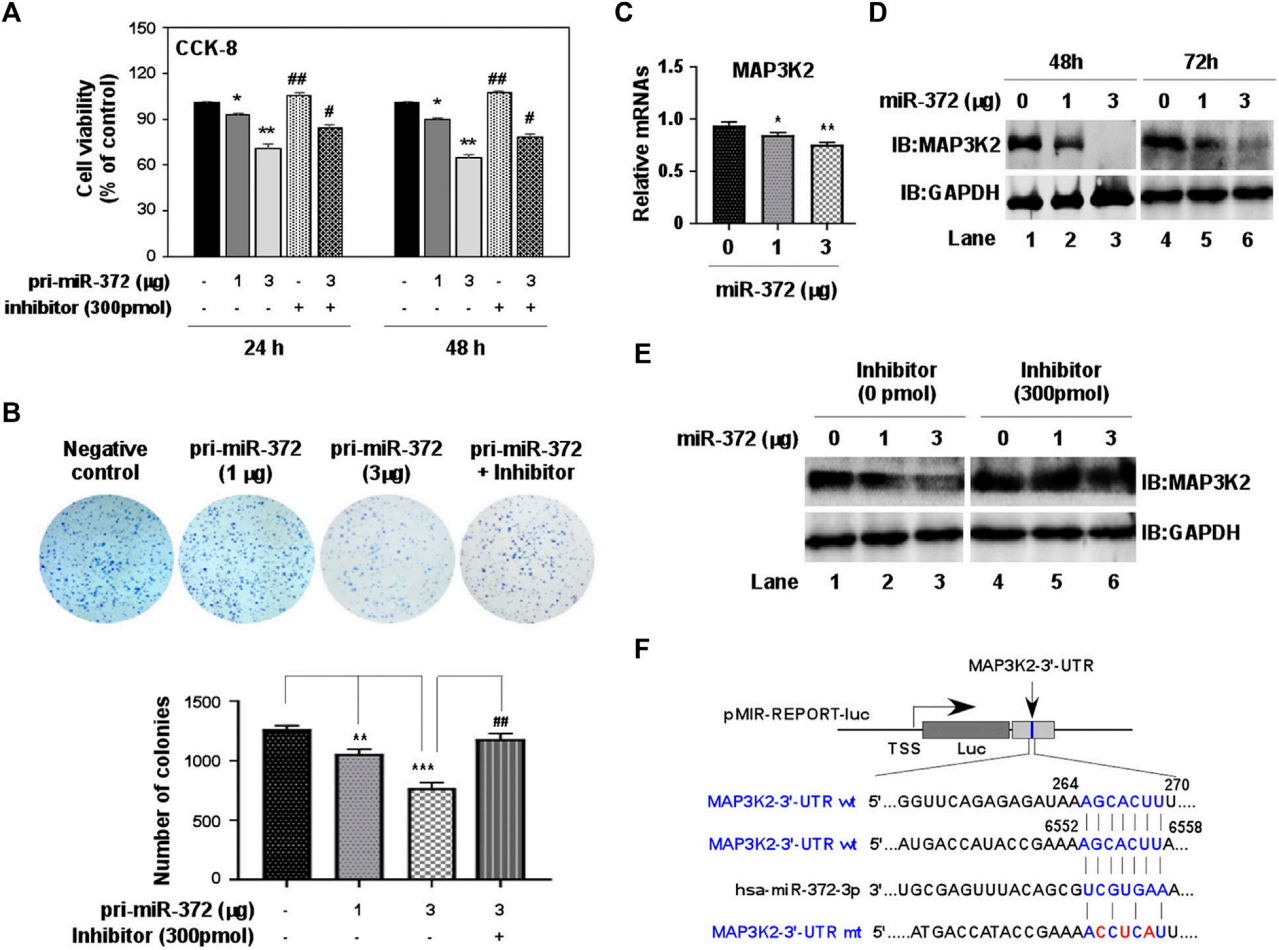
miR-372-3p suppressed cell proliferation in SW480 colon cancer cells, and also negatively regulated MAP3K2 expression. **(A)** SW480 cell proliferation was inhibited by miR-372-3p mimics. SW480 cells were transiently transfected with miR-372-3p mimics in the presence or absence of miR-372-3p inhibitors. The cell viability was then detected using a CCK-8 assay kit at 24 and 48 h. Data are presented using mean ± standard deviation values. *p < 0.05, **p < 0.001, vs. the vector group, ^#^ *p* < 0.05, ^##^ *p* < 0.01, vs. the miR-372-3p 3 µg group (Mann Whitney test). **(B)** Colony-formation assay. The colony-formation ability of SW480 cells was analyzed with a colony formation assay (upper), and the quantified numbers of colonies for each group are displayed as a bar graph (lower). Data are presented using mean ± standard deviation values. **p < 0.01, ***p < 0.001, vs. The vector group, ^##^ *p* < 0.01, ^###^ *p* < 0.001, vs. the miR-372-3p 3 µg group (Mann Whitney test). **(C,D)** Effects of miR-372-3p on the MAP3K2 expression in SW480 cells. Cells were transfected with miR-372-3p mimics (0, 1, 3 µg). MAP3K2 mRNA was detected using RT-qPCR (at 48 h) **(C)**, and the protein levels were analyzed with western bloting (at 48 and 72 h) approach **(D)**. GAPDH was used as an internal control. **(E)** The MAP3K2 protein level reduced by miR-372-3p was restored by co-transfection with miR-372-3p inhibitors in SW480 cells. **(F)** Binding sites of miR-372-3p on the MAP3K2 3′-UTR. The 3′-UTR fragments of human MAP3K2 (+21 to +472 bp, +6394 to +6656 bp) were cloned downstream of the luciferase between the MulI and HindIII sites.

Since the expression of miR-372-3p was significantly different between colon cancer and normal tissues, it is necessary to know whether miR-372-3p can affect the growth and proliferation of colon cancer cells. To do that, a 277-bp DNA fragment carrying pri-miR-372-3p was inserted between the XhoI and BamHI sites in the pmR-mCherry vector (Supplementary Figure S1A). The expression of pri-miR-372-3p was confirmed by transfecting pmR-pri-miR-372-3p plasmids in SW480 colon cancer cells, and this expression was effectively inhibited by co-transfecting with miR-372-3p inhibitors in a dose-dependent manner (Supplementary Figure S1B). After confirming the expression of miR-372-3p, CCK-8 and MTT assays were tested to assess the effect of miR-372-3p mimics on cell proliferation. As shown in Figure 5A, a significant reduction in cell viability was observed at 24 (*p < 0.05) and 48 (**p < 0.01) hours after miR-372-3p mimics application compared to empty vector transfected cells. Simultaneously, this inhibition was effectively restored by adding miR-372-3p inhibitors (#*p* < 0.05). Similar results were obtained in MTT [3-(4,5-Dimethylthiazol-2-yl)-2,5-diphenyltetrazolium bromide] assays (Supplementary Figure S1C). Consistent with this, colony formation results also confirmed that miR-372-3p mimics can inhibit SW480 cell proliferation. The clonogenic ability of SW480 cells was suppressed by transfecting with increasing amounts of miR-372-3p, and this effect was antagonized by inhibitors (Figure 5B, upper). The quantified number of colonies in each group is shown in Figure 5B (lower).

The UALCAN website provides publicly available cancer OMICS data, including from the cancer genome atlas (TCGA), MET500, clinical proteomic tumor analysis consortium, and children’s brain tumor tissue consortium, and can obtain valuable information and data on genes/targets of interest. Supplementary Figure S1D shows a graph downloaded from this website revealing the effect of MAP3K2 expression on the survival of patients with colon adenocarcinoma (http://ualcan.path.uab.edu/cgibin/TCGAsurvival1.pl?genenam MAP3K2&ctype=COAD). Although there was no significant difference in the survival rate between groups with high (*n* = 71) and low (*n* = 208) expression of MAP3K2, the survival period of the low-expression group (>12 years) was obviously longer than that of the high-expression group (<7 years), suggesting that the suppression of MAP3K2 expression in colon cancer may be beneficial to improve the survival rate of patients. In our experiments, overexpression of increasing amounts of miR-372-3p dose-dependently suppressed MAP3K2 expression in both mRNA (Figure 5C) and protein (Figure 5D) levels in SW480 cells. However, co-transfection of miR-372-3p and its inhibitors completely inhibited miR-372-3p mediated reduction of MAP3K2 (Figure 5E), suggesting that miR-372-3p may target and regulate MAP3K2 expression.

**FIGURE 6:**
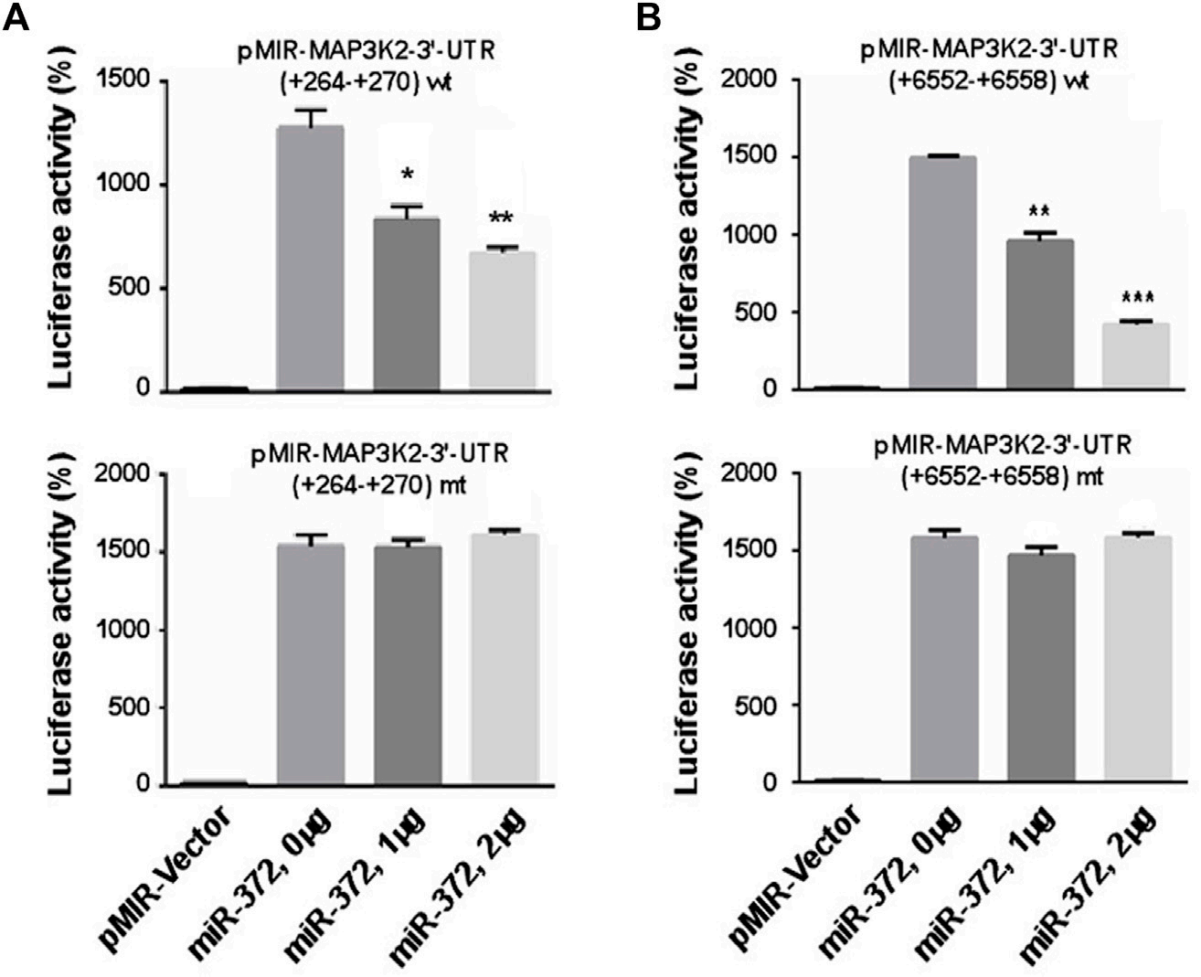
miR-372-3p modulated the expression of MAP3K2 by targeting its 3′-UTR in SW480 colon cancer cells. **(A,B)** Relative luciferase activities of pMIR-MAP3K2 3′-UTR wild type (wt) and mutants (mt) were detected in pMIR-Vector and miR-372-3p mimics (0, 1, and 2 µg) groups. *p < 0.05, **p < 0.01, ***p < 0.001, vs. vector group (Mann Whitney test). Three biological replicates were conducted.

To validate the predicted MAP3K2 that was actually suppressed by miR-372-3p in colon cancer cells, we constructed two luciferase reporter plasmids containing the miR-372-3p target sites (264–270 and 6552–6558) in the MAP3K2 3′-UTR region (Figure 5F). The 3′-UTR luciferase activity of MAP3K2 was significantly repressed by miR-372-3p mimics (Figures 6A,B, upper). Also, while the miR-372-3p target sites in the 3′-UTRs linked to the luciferase reporter were mutagenized, both mutant sites lost their response to miR-372-3p (Figures 6A,B, lower), indicating the site-specificity of the repression.

## Discussion

In this study, we observed that miR-372-3p is less strongly expressed in patients with colon cancer, and this pattern was associated with highly expressed MAP3K2 involved in the regulation of MAP kinases and other signaling pathways (Uhlik et al., 2004; Cuevas et al., 2007). It is well known that the mitogen-activated protein kinase-like protein (MAPK) signaling pathways regulate many cellular processes, such as survival, proliferation, differentiation, migration, and apoptosis. Three of the major MAPK pathways are extracellular regulated kinases 1 and 2, c-Jun-N-terminal kinases (JNKs), and p38 (Qi et al., 2005). Using a candidate gene approach and data on the risk of colon and rectal cancer from population-based case–control studies, Slattery et al. evaluated genetic variations in MAPK pathways, and found that both p38 and JNK were highly expressed in colonic adenomatous polyps; however, the ERK-signaling pathway was more closely associated with rectal cancer given the number of genes in this pathway associated with rectal cancer but not colon cancer (Slattery et al., 2012), suggesting that the MAPK signaling pathway may respond differently in distinct tumors according to the various molecular mechanisms.

MAPK signaling pathway activation is frequently reported in certain carcinoma, (Guo et al., 2018), therefore, inhibition of MAPK signaling pathway activation may be beneficial in cancer therapy. Genome-wide analysis showed that MAP3K2 can act as stress-activated protein kinase in MAPK signaling pathway and may be involved in the regulation of MAPK signaling pathway in cancer deterioration (Pan et al., 2017). In lung cancer tissues, higher expressed MAP3K2 was confirmed. While knockdown of MAP3K2 by siRNA inhibited lung cancer cell proliferation, migration and invasion *in vitro*. Furthermore, miR-186 can suppress cell proliferation and metastasis through targeting MAP3K2 in non-small cell lung cancer (Huang et al., 2016). Subsequent research also confirmed that both miR-302 (Wang et al., 2019) and miR-299-3p (Liu et al., 2020) bind to the complementary sequences of the MAP3K2-3′-UTR, and thereby inhibit cell proliferation by downregulating MAP3K2 in HepG2/SMMC-7721 hepatocellular carcinoma and lung squamous cell carcinoma, respectively. Thus, inhibiting MAP3K2 in certain cancers may play a role in the treatment of cancer.

In our experiments, the higher expression of MAP3K2 in patients with colon cancer was negatively correlated with the miR-372-3p expression, indicating that MAP3K2 may be a potential target of miR-372-3p. However, although the target relationship between miR-372-3p and MAP3K2 was observed, it cannot be determined that miR-372-3p directly regulates MAP3K2, because a single miRNA usually targets multiple potential protein-coding genes (Vincent et al., 2014). Therefore, experimental studies in cells may help to overcome the above limitations. As mentioned before, miR-372 is considered to have dual roles in cells, where it can serve as a tumor-suppressor or an oncogene. In our experimental conditions, lower expression of miR-372-3p was observed in patients with colon cancer. This seems to be contrary to the reported high expression of miR-372 in colorectal cancer. Although we cannot rule out the possibility of discrepancies in results due to the limited number of tissue samples, based on the published literatures, at least the tissue types, tumor progression, the individual differences of patients with cancer, and the different types of genes interacting with miR-372 may be related. However, it is certain that the colorectal samples from patients without liver metastasis expressed miR-372 at a significantly lower level than those from patients with liver metastasis (Yamashita et al., 2012), suggesting that there may be different molecular mechanisms in colorectal cancer cells that are prone to distant metastasis. We also confirmed that mimicking the increased miR-372-3p levels in colon cancer SW480 cells inhibited cell proliferation. Consistent with this, significant low expression of miR-372 was found more frequently in patients with elevated cell proliferation marker Ki67. Importantly, increased miR-372-3p mimics suppressed MAP3K2 in both mRNA and protein levels in SW480 cells in a dose-dependent manner, and this effect was blocked by miR-372-3p inhibitors, prompting the idea that miR-372-3p inhibits the proliferation of SW480 cells by inhibiting the MAPK signaling pathway by targeting MAP3K2.

## Conclusion

In summary, restoration of the expression level of miR-372-3p inhibits the MAPK signal pathway by targeting MAP3K2. This finding not only uncovers the potential molecular mechanism underlying the miR-372-3p inhibition of colon cancer cell growth by targeting MAP3K2 but also opens up a new approach for the development of a therapeutic strategy for colon cancer.

## Data Availability

The original contributions presented in the study are included in the article/Supplementary Material, further inquiries can be directed to the corresponding authors.

## Supplementary Material

Supplementary Figure S1
**(A)** The diagram of miR-372-3p expression plasmid construction. **(B)** Confirmation of miR-372-3p expression in SW480 cells. Cells were transfected with 0, 1, or 3 µg of mR-miR-372-3p plasmids in the presence or absence of 300 pmol of miR-372-3p inhibitors. Then, 48 h later, miR-372-3p levels were measured by qPCR. Data are presented using mean ± standard deviation values. ***p* < 0.01, ****p* < 0.001, Mann Whitney test was performed between miR-372-3p and miR-372-3p + inhibitor cells. **(C)** MTT assay. SW480 cells were transiently transfected with miR-372-3p mimics in the presence or absence of miR-372-3p inhibitors. The cell viability was then detected using a MTT assay kit at 24 and 48 hours. Data are presented using mean ± standard deviation values. **p* < 0.05, ***p* < 0.001, vs. the vector group, ^#^ *p*<0.05, ^##^ *p*<0.01, vs. the miR-372-3p 3 µg group (Mann Whitney test). **(D)** Survival probability of MAP3K2 in colon adenocarcinoma (COAD) (Ualcan database, http://ualcan.path.uab.edu/analysis.html).

## Abbreviations

HDAC4, histone deacetylase 4; HRE-2, hypoxia response element-2; LATS2, Large tumor suppressor homolog 2; MAP3K2, mitogen-activated protein kinase kinase kinase 2 (MEKK2); MAPK, mitogen-activated protein kinase-like protein; MLH1, mutL homolog 1; MSH2, mutS homolog 2; p21, cyclin dependent kinase inhibitor 1A (CDKN1A); PMS2, mismatch repair system component 2; Wee1, WEE1 G2 checkpoint kinase.
