# Supplementary material for: MiR-372-3p Functions as a Tumor Suppressor in Colon Cancer by Targeting MAP3K2
Source: Front Genet. 2022 Mar 30;13:836256. doi: 10.3389/fgene.2022.836256 (PMC9006175; doi:10.3389/fgene.2022.836256)
Supplement: Supplementary file 1 [file Table1.DOCX]

**Table S1 Relationship between miR-372 expression (qPCR) and clinicopathological characteristics of colon cancer.**

| Factor | Case  (n) | Normal  Mean ± SEM  (2^-ΔΔCT^ values) | Cancer  Mean ± SEM  (2^-ΔΔCT^ values) | *p*-value  nor vs. can | | Significantly different |
| --- | --- | --- | --- | --- | --- | --- |
| all  Age (years)  ≤ 60  > 60  Gender  male  female  Pathological stage  pT2  pT3  LN metastasis  N0  N1  N2 | 45  21  24  24  21  12  33  21  16  8 | 0.008478 ± 0.003879  0.009475 ± 0.004485  0.008415 ± 0.003175  0.008753 ± 0.003885  0.009089 ± 0.003863  0.01127 ± 0.003229  0.008051 ± 0.003736  0.008985 ± 0.004357  0.008716 ± 0.003856  0.009058 ± 0.002459 | 0.005606 ± 0.004245  0.006123 ± 0.005086  0.005063 ± 0.00326  0.00451 ± 0.003451  0.006475 ± 0.04644  0.005008 ± 0.004958  0.005757 ± 0.003935  0.00483 ± 0.003737  0.006781 ±0.005324  0.005216 ± 0.002531 | | 0.0002105  0.00021  0.000948  0.000306  0.07765  0.001972  0.019734  0.002428  0.28036  0.007186 | *P*<0.001  *P*<0.001  *P*<0.001  *P*<0.001  NS  *P*<0.01  *P*<0.05  *P*<0.01  NS  *P*<0.01 |

LN, Lympho node; NS, no significant difference. All qPCRs were performed in three independent experiments with three

replicates per group. Statistical differences between two groups were analyzed using the Mann Whitney test.
