# Supplementary material for: MiR-372-3p Functions as a Tumor Suppressor in Colon Cancer by Targeting MAP3K2
Source: Front Genet. 2022 Mar 30;13:836256. doi: 10.3389/fgene.2022.836256 (PMC9006175; doi:10.3389/fgene.2022.836256)
Supplement: Supplementary file 2 [file Table2.DOCX]

**Table S2 Relationship between miR-372 expression (qPCR) and IHC parameters of colon cancer.**

| Factor | Case  (n) | Normal  Mean ± SEM  (2^-ΔΔCT^ values) | Cancer  Mean ± SEM  (2^-ΔΔCT^ values) | *p*-value  nor vs. can | | Significantly different |
| --- | --- | --- | --- | --- | --- | --- |
| P53 IHC (%)  Negative  <20%  21~70%  >70%  Ki67 IHC (%)  <60%  >60%  HRE-2 IHC (%)  Negative  Positive (+)  Positive (++)  MLHs/PMS2 IHC (%)  Negative  Positive (<70%)  Positive (>70%) | 6  9  11  19  9  36  23  16  6  5  30  10 | 0.010471 ± 0.004327  0.010052 ± 0.004581  0.007094 ± 0.00339  0.008927 ± 0.003135  0.007446 ± 0.003025  0.009276 ± 0.003981  0.008591 ± 0.003348  0.008827 ± 0.004185  0.010352 ± 0.004547  0.010811 ± 0.0058692  0.0091177 ± 0.003485  0.007336 ± 0.003101 | 0.00347 ± 0.003025  0.004798 ± 0.00512  0.005666 ± 0.002823  0.006514 ± 0.004493  0.003963 ± 0.003161  0.005956 ± 0.04385  0.005534 ± 0.003947  0.006313 ± 0.004827  0.003633 ± 0.00284  0.0037454 ± 0.002387  0.006005 ± 0.004577  0.005123 ± 0.003599 | | 0.01416  0.04607  0.3184  0.06976  0.03873  0.00145  0.008163  0.138  0.06737  0.05625  0.00506  0.1793 | *P*<0.05  *P*<0.05  NS  NS  *P*<0.05  *P*<0.01  *P*<0.01  NS  NS  NS  *P*<0.01  NS |

IHC, immunohistochemistry; Ki-67, marker of proliferation Ki-67; MSHs, MutS-like proteins; PMS2, PMS1 homolog 2,

mismatch repair system component; NS, no significant difference. All qPCRs were performed in three independent experiments

with three replicates per group. Statistical differences between two groups were analyzed using the Mann Whitney test.
