# Supplementary material for: MiR-372-3p Functions as a Tumor Suppressor in Colon Cancer by Targeting MAP3K2
Source: Front Genet. 2022 Mar 30;13:836256. doi: 10.3389/fgene.2022.836256 (PMC9006175; doi:10.3389/fgene.2022.836256)
Supplement: Supplementary file 3 [file DataSheet1.PDF]

Certificate of English Language Editing

Manuscript Title:

MiR-372-3p functions as a tumor suppressor in colon cancer by targeting MAP3K2

Date of Revision:

January 12, 2022

Abstract:

MicroRNAs (miRNAs) as small non-coding RNA transcripts bind their complementary sequences in the 3'-untranslated region (3'-UTR) of target messenger RNAs (mRNAs) to regulate their expression. It is known that miR-372 belongs to the miR-371-373 gene cluster and has been found to be abnormally expressed in a variety of cancers, but its precise mechanism in cancer remains to be discovered. In this study, miR-372-3p expression was assessed in 153 frozen tissue samples, including primary diagnosed colon cancer and matched normal and adjacent tissues, using real-time quantitative polymerase chain reaction (qPCR). An analysis of qPCR data revealed a significant reduction in miR-372-3p expression (by >2-fold) in colon cancer tissues in 51.5% (34/66) of patients, while, in adjacent tissues, a >2-fold reduction in miR-372-3p expression had already appeared in 11/21 of the patients. Consistent with this, mimicking the increased miR-372-3p levels in SW480 colon cancer cells significantly suppressed cell growth and proliferation. Although no direct correlation was found between the low level of miR-372-3p and certain...

This document certifies that the manuscript listed above was copy edited for English language by LetPub, with regard to grammar, punctuation, spelling, and clarity. All of our language editors are native English speakers with long-term experience in editing scientific and technical manuscripts. We are committed to leveling the playing field for researchers whose native language is not English.

- Documents receiving this certification should be regarded as having undergone professional editorial revision for English language before submission. However, the authors may accept or reject LetPub's suggestions and changes at their own discretion and LetPub does not have editorial control over the submitted documents.
- The language quality of the submitted document is the sole responsibility of the submitting authors subject to those authors' adherence to LetPub's revisions and instruction. LetPub's provision of service does not constitute a guarantee or endorsement of the authors' work herein.
- Neither the research content nor the authors' intended meaning were altered in any way during the editing process.
- If you have any questions or concerns about this edited document, please contact us at support@letpub.com

LetPub
Your Manuscript Editor

LetPub is an author service brand owned and operated by Accdon LLC. Headquartered in the Boston area, we are a full-spectrum author services company with a large team of US-based certified language and scientific editors, ISO 17001 accredited translators, and professional scientific illustrators and animators. We advocate ethical publication practices and are an official member of the Committee on Publication Ethics (COPE).

For more information about our company, services, and partnership programs, please visit www.letpub.com.

© 2022 Accdon, LLC. All Rights Reserved. Tel: 1-781-202-9968 Email: info@accdon.com Address: 400 Fifth Ave, Suite 530, Waltham, MA 02451, United States
